# Retraction Note: Indirubin exerts anticancer effects on human glioma cells by inducing apoptosis and autophagy

**DOI:** 10.1186/s13568-024-01711-6

**Published:** 2024-05-02

**Authors:** Zhaohui Li, Han Wang, Jun Wei, Liang Han, Zhigang Guo

**Affiliations:** 1 Department of Neurosurgery, China-Japan Union Hospital of Jilin University, 130033 Changchun, China; 2 Clinical Laboratory, The Affiliated Hospital of Changchun University of Traditional Chinese Medicine, 130021 Changchun, China; 3 Surgery Department, China-Japan Union Hospital of Jilin University, 130033 Changchun, China; 4 Department of Pathology, China-Japan Union Hospital of Jilin University, 130033 Changchun, China

**Retraction note to: AMB Express (2020) 10:171**

10.1186/s13568-020-01107-2.

The Editor-in-Chief has retracted this Article.

Following publication, concerns were raised about similarities between the published figures and those in previously published articles. Specifically:

Figure 2 A appears to overlap with Fig. 3A from [1];

Figure 2B appears to overlap with Fig. 3B from [2], Fig. 6 from [3] and Figs. 1E and 3H from [4];

Figure 4 A appears to overlap with Fig. 1G F from [5];

Figure 4B appears to overlap with Fig. 6 from [6].

The Editor-in-Chief no longer has confidence in the reliability of the data underlying the findings presented.

None of the authors have responded to any correspondence from the publisher/editor about this retraction.
